# Functional neurological disorders among hospitalized patients in a resource-limited setting: current situation in Colombia and paths to improvement

**DOI:** 10.1007/s10072-025-08416-z

**Published:** 2025-10-01

**Authors:** Daniel S. Marín-Medina, Adriana P. Ortega-Quintero, Paula A. Arenas-Vargas, Juan C. Arias-Botero, Jorge Luis Ramírez-Molina

**Affiliations:** 1 NeuroUnal Research Group, Neurology Unit, National University of Colombia, Carrera 45 N° 26-85, Bogotá, D.C Colombia; 2 Hospital Universitario Nacional de Colombia, Bogotá, Colombia

**Keywords:** Clinical Characteristics, Colombia, Diagnosis, Functional Neurological Disorders, Hospitalization

## Abstract

**Background:**

Functional neurological disorders (FND) are increasingly recognized in various clinical settings. However, early diagnosis and appropriate treatment remain challenging among hospitalized patients.

**Objective:**

To describe the clinical characteristics, diagnostic process, treatment, and outcomes of hospitalized patients with FND and to propose a pathway for improvement.

**Methods:**

We conducted a retrospective chart review of hospitalized adult patients diagnosed with FND between 2020 and 2023 at a tertiary hospital in Colombia. Follow-up was conducted via phone interviews.

**Results:**

Sixty-seven patients were included, 84% female, with a median age of 34 (24–47) years. The most common FND subtypes were weakness (57%) and seizures (49%), with 37% presenting mixed FND. Concomitant symptoms and comorbid medical, neurological, and psychiatric conditions were frequent. Misdiagnosis was common, mostly as stroke or epilepsy/status epilepticus. Only 12% were correctly identified at admission, highlighting a lack of early recognition. The diagnostic process reflected an exclusion-based approach and underuse of appropriate diagnostic criteria for FND. Most patients underwent imaging and additional testing, 16% required extensive workups, and 13% were exposed to potentially harmful interventions. Treatment for FND primarily included outpatient psychotherapy (61%), psychiatric follow-up (66%), and antidepressants (37%). Standard outpatient physiotherapy was only indicated in 21%, and neurology follow-up was not considered in one-third of cases. Among patients contacted by phone (*n* = 51), 37% reported partial or insufficient understanding of the diagnosis.

**Conclusions:**

Limited awareness of FND diagnosis and management is a major factor in misdiagnosis and iatrogenic harm. We proposed an inpatient pathway to improve care for these patients.

**Supplementary Information:**

The online version contains supplementary material available at 10.1007/s10072-025-08416-z.

## Introduction

Functional neurological disorders (FND) are characterized by a heterogeneous group of genuine, involuntary symptoms, including movement disturbances, weakness, sensory deficits, cognitive impairment, and seizure-like events. These conditions share underlying alterations in the neural mechanisms of attentional control, predictive processing errors, and voluntary perception [1, 2] and are influenced by a combination of predisposing factors such as psychological or physical trauma, exposure to diseases, somatic symptoms, psychiatric comorbidities, and other neurobiological factors [3].

The incidence and prevalence of FND range from 10 to 22 and 80 to 140 per 100,000 individuals, respectively [4]. FND is one of the most frequent reasons for consultation and is among the three primary diagnoses in outpatient clinics [5, 6]. In emergency departments, up to a quarter of patients with suspected epileptic seizures present with functional/dissociative seizures (FDS), and 8% of patients in stroke units have an FND [7]. Some of these patients are not initially diagnosed with an FND due to their heterogeneous presentations, similarities to other neurological conditions, and limitations in adequately identifying the positive signs of FND. In many instances, these patients require hospitalization for additional investigations or interventions to exclude potentially life-threatening conditions or other complex diagnoses. This situation raises the potential for misdiagnosis, increased risk of iatrogenic harm, high cost from unnecessary investigations, prolonged hospital stays, and lost opportunities for specific treatment of FND, all of which could lead to worse outcomes [8, 9].

Understanding the specific needs and challenges of hospitalized FND patients could facilitate their early diagnosis, rational use of investigations, and timely treatment initiation. This is particularly relevant in resource-limited settings, such as Colombia. For this reason, this study aimed to describe the clinical presentation, diagnostic work-up, and treatments of hospitalized FND patients and to propose a pathway for resource-limited settings.

## Methods

We conducted a retrospective chart review of adult patients admitted to the hospitalization service of the Hospital Universitario Nacional de Colombia (HUN) in Bogotá, Colombia, between 2020 and 2023. HUN receives only adult patients referred for hospitalization from several clinics in Bogotá and nearby cities, mainly from the emergency department. Patients were covered under Colombia’s contributory health insurance scheme, which includes formally employed individuals and their dependents, primarily from the middle socioeconomic class.

The patients included in this study were previously evaluated by an external emergency physician, internist, or neurologist who decided to continue inpatient management if necessary. Subsequently, patients were admitted to the Department of Internal Medicine, where the attending physician determined the next steps in their management and the need for neurological consultation.

Patients were identified by reviewing the registry of all individuals evaluated by the neurology service during the study period. The inclusion criteria were: 1) adult patients evaluated by a hospital neurologist and 2) a probable diagnosis of FND with sufficient data in the clinical records. Patients with a doubtful diagnosis of FND during hospitalization were excluded.

Clinical records were obtained from the patient registry. Each record was reviewed to determine if it met the inclusion criteria and to extract data from the free-text sections. All eligible patients were contacted by telephone during February 2024, and a standardized questionnaire was administered (Supplemental Table 1).

**Table 1:** Demographic and clinical characteristics of hospitalized FND patients

Age, years, median (IQR)	34 (24–47)
Sex, *n* (%)	
Female	56 (84)
Origin, *n* (%)	
Urban	56 (84)
Education level, *n* (%)	
Elementary	10 (15)
High school	45 (67)
University	12 (18)
Work status, *n* (%)	
Employed	28 (42)
Previous FND symptoms, *n* (%)	28 (42)
Medical comorbidity, *n* (%)	33 (49)
Neurological comorbidity, *n* (%)	27 (40)
Epilepsy	9 (13)
Migraine	13 (19)
Other	8 (12)
Psychiatric comorbidity^a^, *n* (%)	39 (58)
Depression	7 (10)
Anxiety	11 (16)
Anxiety and depression	4 (6)
Adjustment disorder	4 (6)
PTSD	2 (3)
Maladaptive personality traits	15 (22)
Psychological aspects, *n* (%)	
History of trauma	10 (15)
Recent stressful event^b^	38 (57)

^a^Includes pre-existing and newly diagnosed psychiatric comorbidity during the hospitalization

^b^Reported by the patient

*FND* functional neurological disorder, *PTSD* post-traumatic stress disorder

The following variables were collected:

1. Sociodemographic: age, sex, origin (rural or urban), educational level, and employment status.
2. Clinical: initial neurological symptoms at arrival and concomitant symptoms; medical, neurological, and psychiatric comorbidities; history of violence or trauma; diagnosis suspected by the first-contact physician; and number of days of hospital stay.
3. Features of FND: findings of inconsistency (variability, distractibility, suggestibility, or specific signs such as Hoover's sign) and incongruence; Video-EEG (electroencephalography) descriptions in patients with FDS.
4. Additional investigations and interventions: type and number of studies performed; pharmacological treatment; orotracheal intubation; and extensive diagnostic work-up (defined as more than one week of hospital stay due to the requirement of several investigations).
5. Discharge indications: follow-up by a medical specialist, psychotherapy, occupational or physical therapy.
6. Follow-up: patient-reported improvement of the FND symptoms, change in diagnosis, and patient perception of the FND explanation.

### Statistical analysis

Continuous variables were analyzed by calculating the mean, standard deviation, median, and interquartile range, depending on whether the variable followed a normal distribution. Categorical variables were analyzed by absolute frequencies and proportions. Data analysis was performed using the R statistical software.

This research was approved by the HUN research ethics committee, according to resolution 8430 of 1993 issued by the Colombian Ministry of Health, and with the Declaration of Helsinki.

## Results

### Sociodemographic characteristics

A total of 67 patients were included, with median age of 34 years (24–47), 56 were female (84%), 56 lived in urban locations (84%), 45 completed high school (67%), and 39 were unemployed (58%) (Table 1).

### Clinical characteristics at first evaluation

A previous stressful event was self-reported in 38 patients (57%), and a previous functional neurological symptom in 28 (42%). The initial symptoms were weakness and seizure/syncope-like events in 38 (57%) and 33 (49%) patients, respectively. Isolated FND was the leading presentation in 42 patients (63%), and the remaining exhibited mixed FND. Concomitant symptoms were frequent (74%) (Table 2).

**Table 2:** Initial symptoms of hospitalized FND patients

FND symptoms^a^, *n* (%)	
Hemiparesis	16 (24)
Paraparesis	4 (6)
Quadriparesis	7 (11)
Isolated facial paresis	2 (3)
Another type of paresis	9 (13)
Seizure-like	28 (42)
Syncope-like	7 (11)
Hemi-sensory alteration	9 (13)
Another sensory pattern	5 (8)
Movement	9 (13)
Gait	3 (4)
Speech/voice	5 (8)
Number of FND symptoms, *n* (%)	
1	42 (63)
≥ 2	25 (37)
Concomitant symptoms^a^, *n*(%)	
Headache	29 (43)
Chest pain	10 (15)
Another type of pain	7 (11)
Vertigo	4 (6)
Shortness of breath	4 (6)
Number of concomitant symptoms, *n* (%)	
1	37 (55)
≥ 2	13 (19)

^a^Note that some patients had more than one symptom

*FND* functional neurological disorder

Medical (49%) and neurological comorbidities (40%) were frequent. Pre-existing psychiatric comorbidity was reported in 18 patients (27%). After formal evaluation by a psychiatrist during the hospitalization, a new psychiatric diagnosis was made in 21 patients (31%). After psychological assessment, 10 patients (15%) reported a history of trauma, including various forms of physical, psychological, sexual, and armed conflict-related violence.

### Initial diagnostic workup

After evaluation by the first-contact physician, only 8 patients (12%) were suspected of having FND. In most cases, there was an initial provisional diagnosis of a neurological condition such as stroke (25%), epileptic seizures (11%), syncope (6%), status epilepticus (13%), a life-threatening cause related to secondary headache (8%), and, less frequently, infections or neuromuscular causes. For this reason, additional investigations were performed in 58 patients (87%) before a neurology consultation. Imaging and other diagnostic tests were frequently indicated (Table 3). An extensive diagnostic work-up was required in 16% of patients.

**Table 3:** Diagnostic work-up of hospitalized FND patients

FND suspected by first contact physician, *n* (%)	8 (12)
Other diagnoses considered, *n* (%)	
Stroke or TIA	17 (25)
Syncope or cardiovascular	7 (10)
Headache with red flags	5 (8)
Epileptic seizure	7 (11)
Status epilepticus	9 (13)
Other^a^	16 (24)
Initial orders, *n* (%)	
Additional investigations	58 (87)
Observation or neurology consultation	9 (13)
Imaging and additional studies, *n* (%)	
Head CT	34 (51)
Brain MRI	51 (76)
Video EEG	18 (28)
EMG + NC	6 (9)
Lumbar puncture	4 (6)
Other	2 (3)
Positive signs of FND, *n* (%)^b^	
Inconsistency	37 (55)
Suggestibility	8 (12)
Distractibility	17 (25)
Variability	22 (32)
Incongruence	35 (52)
Other reasons for the FND diagnosis, *n* (%)	
Normal studies	6 (9)
Psychiatric comorbidity	4 (6)
Recent stressful event	8 (12)
*La belle indifférence*	2 (3)
Spontaneous recovery	3 (4)

^a^Includes: myopathy, myelopathy, neuropathy, radiculopathy, CNS infectious disease, and dyskinetic disorders

^b^Note that most patients could have more than one positive sign or feature of FND

*CT* computerized tomography, *CNS* central nervous system, *EEG* electroencephalogram, *EMG* + *NC* electromyography and nerve conduction, *FND* functional neurological disorder, *MRI* magnetic resonance imaging, *TIA* transient ischemic attack

### FND diagnosis

FND was considered for the first time by the neurologist in 48 patients (72%). Some findings from the history, such as psychiatric comorbidity (6%), a recent stressful event (12%), and *la belle indifférence* (3%), were reported as arguments for the diagnosis (Table 3). Positive signs of inconsistency were described in 37 patients (55%), while incongruence and normal studies were the main reasons for the diagnosis in 35 (52%) and 6 (9%) patients, respectively. All 28 patients with FDS underwent video-EEG recording. Signs of automatic-voluntary performance, such as the Hoover sign, were described in 8 patients (24%) with weakness (Table 4).

**Table 4:** Positive signs in the diagnosis of hospitalized FND patients

Functional/dissociative seizures, *n* (%)	*n* = 28
Closed eyes	8 (29)
Preserved awareness	7 (25)
Absence of postictal state	7 (25)
Pelvic or axial movements	3 (11)
Arch posture	3 (11)
Asynchrony	3 (11)
Ictal crying	3 (11)
Always witnessed	2 (7)
Side-to-side head movement	1 (4)
Initial hyperventilation	1 (4)
Weakness, *n* (%)	*n* = 34
Motor impersistence	21 (62)
Hoover test	8 (24)
Sensory disturbance, *n* (%)	*n* = 14
Bowlus-Currier maneuver	1 (7)
Gait disorder, *n* (%)	*n* = 9
Astasia/abasia	3 (33)
Ice walk pattern	6 (9)

*FND* functional neurological disorder

### Treatment and discharge indications

Of the patients considered for stroke, antiplatelets and preventive medications were indicated in 12 (18%), and 3 (4%) underwent intravenous thrombolysis; meanwhile, 12 patients (18%) with suspected epilepsy received oral or intravenous antiseizure medications, and 9 (13%) were intubated and transferred to the intensive care unit. Following the diagnosis of FND, these treatments were discontinued as clinically appropriate. After psychiatric assessment, 25 (37%) and 4 (6%) were started on antidepressant or antipsychotic medications, respectively (Table 5).

**Table 5:** Treatment, discharge indications, and outcomes of hospitalized FND patients

Pharmacological treatment, *n* (%)	
ASA and statins	12 (18)
Antiseizure medications	12 (18)
Antidepressants	25 (37)
Antipsychotics	4 (6)
Other interventions, *n* (%)	
Tracheal intubation	9 (13)
Intravenous thrombolysis	3 (4)
Indications at discharge, *n* (%)	
Follow-up by neurologist	46 (69)
Follow-up by a psychiatrist	44 (66)
Psychotherapy	41 (61)
Physiotherapy or OT	14 (21)
Ambulatory test	3 (5)
None	3 (5)
Patient perception of the FND explanation, *n* (%)	*n* = 51
Good	32 (63)
Partial	15 (29)
Insufficient	4 (8)
Patient-reported improvement, *n* (%)	*n* = 51
Total	24 (47)
Partial	19 (37)
Unchanged	1 (2)
Worsened	7 (14)

*ASA* acetylsalicylic acid, *FND* functional neurological disorder, *OT* occupational therapy

The median length of stay was 5 days (4–7), with a minimum of 2 and a maximum of 60 days. At discharge, 69% of the patients were referred to a neurologist, 66% to a psychiatrist, 61% to a psychotherapist, and only 21% to a physical or occupational therapist. Outpatient testing was ordered for 5% of patients, and 5% received no follow-up orders.

### Follow-up

After the hospitalization, 51 patients (76%) were contacted via phone interviews. Non-responders consisted mainly of unanswered calls, and only one patient refused to participate. The main demographic and clinical characteristics between responders and non-responders were not statistically significant.

The average time post-discharge until the phone interview was 27.7 ± 11.9 months (minimum 3.3 and maximum 62.9 months). Patient perception of explanation of FND during the hospitalization and patient-reported improvement are summarized in Table 5. Only one patient was reclassified with a diagnosis different from FND.

## Discussion

In this cohort of hospitalized patients with FND in Colombia, common misdiagnoses, delayed recognition, a diagnostic approach based on exclusion, potentially harmful treatments, and a lack of appropriate treatment modalities for FND were frequently observed. These findings reflect current gaps in the inpatient management of FND, particularly in resource-limited settings, and underscore the need for implementing structured inpatient pathways and increasing FND awareness among the various healthcare providers involved in the care of these patients.

Our patients'demographic and clinical characteristics were similar to those of FND patients in different clinical settings [10–12], although our cohort was younger. Regarding clinical manifestations, weakness and seizure-like events (including syncope) were the main clinical phenotypes. Weakness was kept from the functional movement disorders (FMD) subtype because it is relevant in the acute scenario [13]. Interestingly, sensory symptoms were highly prevalent, probably accounting for their coexistence with weakness [14].

In this study, the high frequency of multiple FND, comorbidities, and additional symptoms likely made the initial diagnosis more challenging [15]. These characteristics in individuals referred to hospitalization settings, where there is a priority to exclude potentially life-threatening conditions, could explain the first diagnostic considerations, such as stroke or status epilepticus, the need for additional investigations or interventions, and the low suspicion of FND at first arrival. However, a lack of appropriate knowledge and skills in identifying FND may also explain these findings, which highlights the need to incorporate FND in the radar of first-contact physicians [16] to contextualize investigations better and prevent iatrogenic harm derived from misdiagnosis and invasive testing or interventions, which have been associated with the perpetuation of FND, high cost and worse outcomes [9].

Regarding FND diagnosis by neurologists, there was significant reliance on some historical findings (e.g., psychiatric comorbidity) and physical findings (e.g., *la belle indifférence*), which are poorly specific [17, 18]. Despite this, most of the patients diagnosed with FND were reported as having both features of inconsistency and incongruence, which, together with the low change of diagnosis in the follow-up (1/51), could indicate good diagnostic performance but highlight the need for updating FND knowledge among neurology consultants, and better education for neurology trainees [19]. Llano-Piedrahita et al. (unpublished data) conducted a survey on FND training during neurology residency in Colombia, which showed that nearly half of the residents and neurologists (*n* = 89) had not received education in the diagnosis and treatment of FND. These gaps in neurology training and the low awareness among healthcare providers may have contributed to the observed delays in diagnosis, misdiagnosis, and mismanagement, and underscore the urgent need to include FND in neurology residency and medical school curricula.

It has been shown that the prognosis of FND is equal to or worse than other prevalent neurological diseases [20], and delayed or inconsistent care pathways could lead to missed therapeutic opportunities, perpetuation of the FND, and recurrent cycles of healthcare utilization [10, 21, 22]. In our study, FND patients were not given inpatient treatment, but most were referred to outpatient standard treatment modalities. Although this could be reasonable for the limitations in resources and time, it is preferable for patients to start treatment as soon as possible, even in hospitalization, and to be treated by allied health professionals with training in FND-specific modalities [23]. The fact that many patients were referred solely to psychiatric follow-up or psychotherapy, as well as the lack of follow-up in a considerable proportion of patients and the low referral to physiotherapy, especially given the frequency of functional weakness, may be explained by the still predominant conception of FND as a mind-only or'psychogenic'condition. This highlights the need for neurologists to update their understanding of FND and to commit to the continued follow-up of these patients [24].

**Fig. 1:**
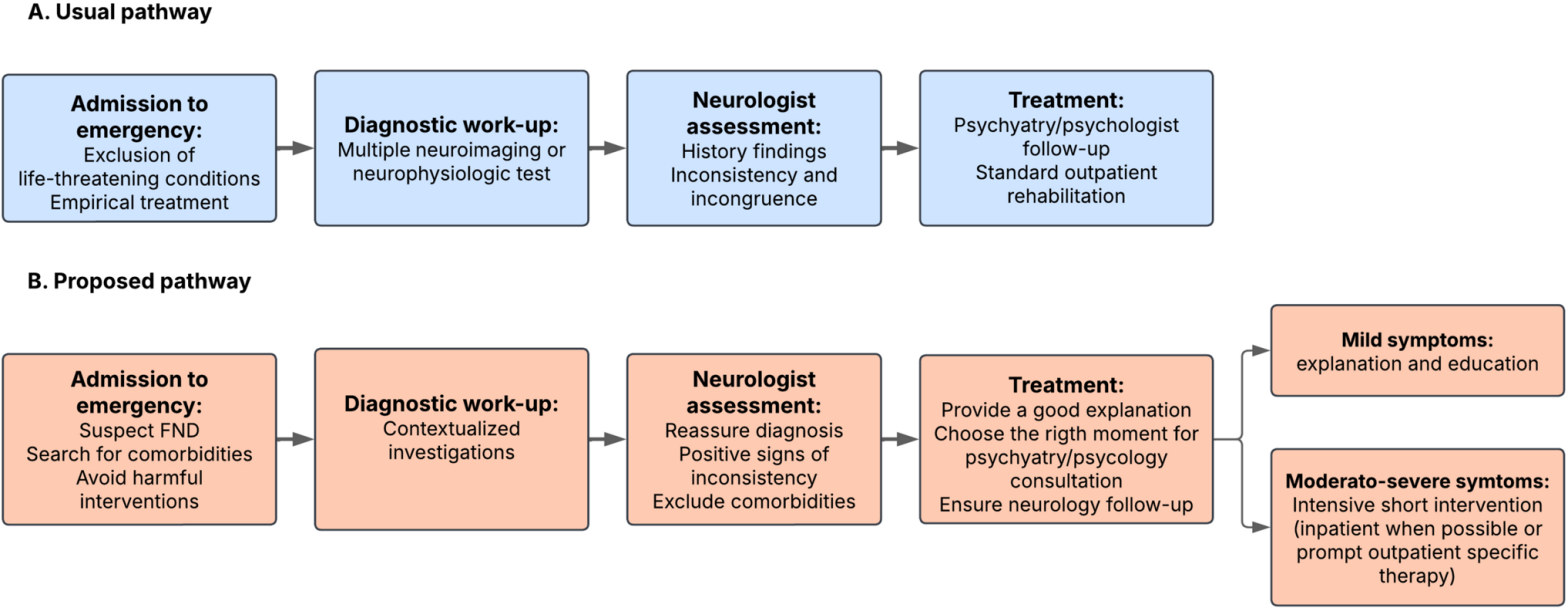
Usual and proposed pathway for hospitalized patients with FND in resource-limited settings

Given this study's findings, we propose an alternative pathway for FND patients in hospitalization (Fig. 1). Regarding diagnosis, after admission to the emergency department, first-contact physicians should be alert for FND in cases of inconsistent clinical presentation while maintaining a low threshold for identifying comorbidities. Investigations should be contextualized to the patient's symptoms, and potentially harmful interventions must be avoided.

Neurologists must be familiar with FND diagnostic criteria and avoid overreliance on poorly specific history, imaging, or physical findings. They should lead the multidisciplinary team, incorporating rehabilitation professionals when possible. Inpatient psychiatry or psychology consultation should be considered only in cases of excessive psychiatric comorbidities or significant perpetuating factors interfering with recovery, as patients may be reluctant to be initially labeled with a “psychogenic” disorder. For patients with mild symptoms, explaining the diagnosis and providing educational resources may be sufficient [13, 25]. For patients with moderate or severe symptoms, it is reasonable to consider a short, intensive inpatient intervention (e.g., psychotherapy for FDS or physiotherapy for FMD), especially when there is a high likelihood that the patient could miss the opportunity of being treated [26]. If inpatient treatment is not an option, an outpatient short intervention, initiated as soon as possible and complemented by neurology follow-up, could be considered. Providing a structured work plan and recommendations, or establishing direct communication with the therapist, may improve treatment outcomes [27].

Our study has several limitations, most of them due to its retrospective nature. It is possible that many patients who attended emergency services were not hospitalized after being diagnosed with FND due to mild symptoms, referral to outpatient management, or administrative barriers. This may have introduced selection bias. Another potential source for selection bias was the high rate of psychiatric comorbidity in the patients, which may have made them more likely to be hospitalized or easily identified as having FND. Since we extracted information from the free-text sections of the clinical records, we may have underestimated the proportions. In the assessment of outcomes, differences in follow-up timing may have affected recall or outcome reporting. Although no statistically significant differences were found between responders and non-responders, and non-responders were simply those who could not be reached, we cannot determine whether the outcomes and follow-up information of the 24% who did not respond to the phone interview were comparable to those of the responders.

## Conclusions

Hospitalized patients with FND have clinical characteristics similar to patients with FND in other settings but face particular challenges for diagnosis and treatment in resource-limited settings. Changing the approach to these patients and improving the training of the health providers involved in their care might lead to early identification and better formulation of management strategies, which can ultimately contribute to better outcomes, lower costs, and more efficient resource allocation.

## Supplementary Information

Below is the link to the electronic supplementary material.

Supplementary file1 (DOCX 138 KB)

## Data Availability

The data that support the findings of this study are available upon reasonable request.
