# Supplementary material for: Functional neurological disorders among hospitalized patients in a resource-limited setting: current situation in Colombia and paths to improvement
Source: Neurol Sci. 2025 Oct 1;46(12):6843–50. doi: 10.1007/s10072-025-08416-z (PMC12678571; doi:10.1007/s10072-025-08416-z)
Supplement: Supplementary file 1 — Supplementary file1 (DOCX 138 KB) [file 10072_2025_8416_MOESM1_ESM.docx]

**Supplemental material**

**Supplemental Table 1.** Structured Phone Interview Questionnaire.

| **No.** | **Questionnaire Item** |
| --- | --- |
| 1 | Please rate your perception of the diagnosis you received during your hospitalization*   - Good: You were given sufficient information about your diagnosis. A specific name was provided for your neurological symptoms, the diagnostic process was explained, and the necessary rehabilitation plan was described. - Partial: You were given some information about your diagnosis, but a specific name was not provided for your neurological symptoms, the diagnostic process was only superficially explained, or the rehabilitation plan was not described in detail. - Insufficient: You were not given information about your diagnosis. A specific name was not provided for your neurological symptoms, the diagnostic process was not explained, and the rehabilitation plan was not mentioned. |
| 2 | Please rate the improvement you have experienced so far in the symptoms for which you were hospitalized:   - Total: I have experienced complete improvement in my symptoms. - Partial: I have experienced some improvement in my symptoms. - Unchanged: I have not experienced any improvement or worsening in my symptoms. - Worsening: I have experienced a worsening of my symptoms. |
| 3 | Since your hospitalization, has a neurologist given you a different diagnosis for your neurological symptoms? |

* Patients were instructed to refer to the hospitalization related to the FND symptoms.
